# The association between *BDNF* Val66Met polymorphism and emotional symptoms after mild traumatic brain injury

**DOI:** 10.1186/s12881-017-0518-0

**Published:** 2018-01-22

**Authors:** Yu-Jia Wang, Kai-Yun Chen, Li-Na Kuo, Wen-Chang Wang, Yu-Wen Hsu, Henry Sung-Ching Wong, Chien-Min Lin, Kuo-Hsing Liao, Yan-Feng Zhang, Yung-Hsiao Chiang, Wei-Chiao Chang

**Affiliations:** 1 Program for Neural Regenerative Medicine, College of Medical Science and Technology, Taipei Medical University, Taipei, Taiwan; 2 Department of Pharmacy, Wan Fang Hospital, Taipei Medical University, Taipei, Taiwan; 3 Program for Translational Medicine, College of Medical Science and Technology, Taipei Medical University, Taipei, Taiwan; 4 Department of Clinical Pharmacy, School of Pharmacy, Taipei Medical University, Taipei, Taiwan; 5 Program for Translational Medicine, College of Medical Science and Technology, Taipei Medical University and Academia Sinica, Taipei, Taiwan; 6 Department of Neurosurgery, Shuang Ho Hospital, Taipei Medical University, New Taipei City, Taiwan; 7 Department of Neurosurgery, Wan Fang Hospital, Taipei Medical University, Taipei, Taiwan; 8 HudsonAlpha Institute for Biotechnology, Huntsville, AL USA; 9 Division of Neurosurgery, Department of Surgery, Taipei Medical University Hospital, Taipei, Taiwan; 10 Master Program for Clinical Pharmacogenomics and Pharmacoproteomics, School of Pharmacy, Taipei Medical University, Taipei, Taiwan; 11 Center for Biomarkers and Biotech Drugs, Kaohsiung Medical University, Kaohsiung, Taiwan

**Keywords:** Mild traumatic brain injury, Genetic polymorphism, Brain-derived neurotrophic factor, Anxiety, Depression

## Abstract

**Background:**

Brain-derived neurotrophic factor (BDNF) is one of the most abundant neurotrophins in the adult brain, and it plays important roles in modulating synaptic plasticity and synaptogenesis. This study attempted to elucidate the role of the *BDNF* variant rs6265 in emotional symptoms following mild traumatic brain injury (mTBI).

**Methods:**

To investigate the association between *BDNF* Val66Met polymorphism (rs6265) and emotional symptoms in mTBI patients, we recruited 192 mTBI patients and evaluated their Beck Anxiety Inventory (BAI) and Beck Depression Inventory (BDI) scores in the first and sixth week after mTBI.

**Results:**

The patients carrying the T allele of rs6265 had significantly higher BAI scores in the first week following mTBI. In addition, the patients carrying the T allele also showed higher scores of BDI in the first week. In the gender-specific subgroup analysis, the male patients carrying the T allele of rs6265 had higher scores of both BAI and BDI in the first and sixth week. Meanwhile, female patients carrying the T allele also had significantly higher scores of BDI in the first week following mTBI.

**Conclusions:**

This study provides evidence for the association between the *BDNF* variant rs6265 and emotional symptoms following mTBI.

**Electronic supplementary material:**

The online version of this article (10.1186/s12881-017-0518-0) contains supplementary material, which is available to authorized users.

## Background

Traumatic brain injury (TBI) is a leading cause of death and disability worldwide. The rates of TBI-related emergency department (ED) visits have been estimated to be increased by 70% over the past decade. In 2010, The Center for Disease Control and Prevention (CDC) reported that 2.5 million ED visits, hospitalizations, and deaths are associated with TBI in the United States. Additionally, current data indicate that about 3.2 ~ 5.3 million persons in the United States are living with disabilities related to TBI [1, 2]. TBI is an alteration of the brain’s normal functioning, caused by a blow or external force to the head [3]. The most common causes of TBI include falls, traffic accidents, and assaults [4]. The severity of TBI can be categorized based on the clinical presentation using the Glasgow Coma Scale (GCS), which is to assess three components of the neurologic functions: eye opening, verbal response, and motor response. Patients with overall scores of 3 ~ 8 are classified as severe, those of 9 ~ 12 are moderate, and those of 13 ~ 15 are mild [5]. More than 75% of TBI cases are classified as mild TBI (mTBI) [6]. Although most mTBI cases are expected to fully recover within days to months, some patients still show persistent cognitive, physical, and emotional impairments. Furthermore, psychological and neurological disorders can also develop after an mTBI (e.g., depression, epilepsy, and insomnia). These chronic health effects of mTBI may cause difficulties returning to one’s routine, daily activities, and work [7, 8].

The molecular repair mechanisms following brain injury are complex and multifactorial processes. Recently, researchers have focused on genetic factors that influence the TBI pathophysiology. For example, apolipoprotein E (*APOE*), the gene located at position q13.2 on chromosome 19 is one of the most widely studied genetic factors for posttraumatic recovery [9]. ApoE is a plasma lipoprotein that maintains the integrity of neuronal membranes, and functions in neuronal repair and remodeling. Patients with *APOE4* allele showed a higher risk of poor clinical outcomes following TBI [9–11]. Genetic variants of monoamine oxidase A (*MAO-A*), interleukin 6 (*IL-6*), and catechol-O-methyltransferase (*COMT*) have also been studied [12–15].

Brain-derived neurotrophic factor (BDNF) is one of the most abundant neurotrophins in the central nervous system, and plays important roles in neuronal growth, differentiation, apoptosis, and synaptic plasticity [16–18]. BDNF has been found to associate with cognitive function, personality development, psychiatric disorders, and neurodegenerative diseases [19–21]. Accumulating evidence indicates the involvement of BDNF in the pathophysiology of mood disorders [22–25]. Previous animal study showed that *BDNF* knockdown in the hippocampal subregion produced depression-like behaviors in rats [26]. In addition, the decreased *BDNF* signaling was observed in the subgenual anterior cingulate cortex in major depressive patients [27]. Furthermore, the recent meta-analysis study revealed that the BDNF blood level had significantly difference between the healthy subjects and patients with major depression [28].

The single nucleotide polymorphism (SNP) rs6265 located in the coding region of the *BDNF* gene is one of the most important SNPs. A change of amino acids from valine to methionine (Val66Met) alters the intracellular tracking of pro-BDNF and affects the secretion and neuroplastic effect of the mature BDNF protein [29, 30]. Growing evidence highlights the role of rs6265 in psychiatric disorders, suicidal behavior and the pharmacologic treatment responses [31–34]. Previous studies supported the important role of the rs6265 polymorphism in cognitive performance and executive function following TBI [35, 36]. However, the role of rs6265 in TBI-related emotional symptoms has not been characterized. We therefore hypothesized that rs6265 might also be associated with emotional symptoms following TBI. Self-reporting questionnaires (Beck Anxiety Inventory 〔BAI〕 and Beck Depression Inventory 〔BDI〕) in the first and sixth week were collected to evaluate emotional symptoms. Previous studies found a gender-specific influence of *BDNF* on mood disorders [37–40]. Therefore, we wished to conduct a gender-specific subgroup analysis to find out whether the association between the *BDNF* variant rs6265 and emotional symptoms following mTBI is affected by gender.

## Methods

### Subject recruitment

Patients aged ≥ 20 years who had been diagnosed with mTBI in the emergency department were recruited from three Taipei Medical University (TMU) affiliated hospitals, including TMU Hospital, Wan Fang Medical Center, and Shuang Ho Hospital. The diagnosis of mTBI was determined according to diagnostic criteria established by the American Congress of Rehabilitation Medicine, a GCS score of 13 ~ 15, and a time of loss of consciousness of < 30 min. This study was approved by TMU Joint Institutional Review Board, with the requirement to have written informed consent.

### Self-reporting questionnaires

Emotional symptoms of included patients were evaluated in the first- and sixth-week visits after a diagnosis of mTBI. The BAI and BDI were completed by patients to respectively evaluate anxiety and depression symptoms. The BAI is a 21-item self-reporting inventory to assess the severity of anxiety symptoms. Each item is rated on a 4-point scale from 0 (not at all) to 3 (severely). Total scores range 0~63, with 0 ~ 7 indicating minimal anxiety, 8 ~ 15 mild anxiety, 16 ~ 25 moderate anxiety, and 26 ~ 63 severe anxiety [41]. The BAI has a high internal consistency (Cronbach’s α = 0.92) and a test-retest reliability over 1 week of 0.75 [41]. BDI, a 21-item self-reporting inventory, is to measure cognitive, behavioral, and physiological symptoms associated with depression. Responses are made on a 4-point scale from 0 to 3, with total scores ranging 0 ~ 63. Cutoff ranges are as follows: 0 ~ 9 indicates normal, 10 ~ 18 mild depression, 19 ~ 29 moderate depression, and 30 ~ 63 severe depression. Cronbach’s α has been reported as 0.92, and the test-retest correlation over 1 week as 0.93 [42, 43].

### DNA extraction

DNA was extracted from the whole-blood samples of mTBI patients. Blood cells were first treated with 0.5% sodium dodecyl sulfate (SDS) lysis buffer, and then a proteinase K solution (1 mg/mL) was used for 4 h at 60 °C to digest the nuclear proteins. Total DNA was harvested using a Gentra extraction kit (Qiagen, Valencia, California, USA) followed by 70% alcohol precipitation.

### Genotyping of *BDNF* rs6265

Genotyping for *BDNF* rs6265 (Val66Met) polymorphism was performed using the TaqMan Allelic Discrimination Assay (Applied Biosystems, Foster City, California, USA). A polymerase chain reaction (PCR) used a 96-well micro-plate with the ABI 9700 Thermal Cycler (Applied Biosystems, Foster City, California, USA). The thermal cycle conditions of the PCR were set as follows: denaturing at 95 °C for 10 min, followed by 40 cycles of denaturing at 95 °C for 15 s, and annealing and extension at 60 °C for 1 min. After the PCR, StepOne software (version 2.2.2, Applied Biosystems, Foster City, California, USA) was used to detect and analyze the fluorescence intensity.

### Statistical analysis

R 3.2.0 (http://www.r-project.org) was used for the statistical analyses. A Linear regression model was used for patient characteristics to define the possible confounding factors, including age, gender, Glasgow Coma Scale (GCS), Extended Glasgow Outcome Scale (GOSE), mechanism of injury and current medication use. We analyzed the magnitude of the association between the different genotypes of rs6265 and BAI, BDI scores through a likelihood ratio test in four models (including codominant, dominant, recessive and log-additive model) that implemented in *SNPassoc* package. BAI and BDI scores of each genotype group were presented with mean ± standard error (s.e.). Patients with missing or incomplete data of BAI, BDI and baseline covariates were excluded from the analyses. Statistical significance was considered at *p* < 0.05.

## Results

### Demographics of mTBI patients

In total, we recruited 192 study patients with mTBI to evaluate the emotional symptoms in the first and sixth week after brain injury. Demographic characteristics are summarized in Table 1. Patients age ranged 20 ~ 83 years, with a mean age of 39.3 years. Females accounted for 68.2% (131/192) of total recruited patients. The causes of brain injury were mainly from traffic accidents 55.7% (107/192), falls 30.7% (59/192), and sports-related injuries or workplace accidents 13.5% (26/192). However, some patients were lost to follow-up after the first week’s visit. In total, 103 (54%) study patients had attended the sixth-week’s visit and completed the assessment.

**Table 1:** Characteristics of patients with mild traumatic brain injury (mTBI) in the first and sixth week

Patient characteristic	First week visit (*n* = 192)	Sixth week visit (*n* = 103)
Gender, no. (%)		
Female	131 (68.2)	67 (65.0)
Male	61 (31.8)	36 (35.0)
Age, years^a^	39.3 ± 15.4	40.4 ± 15.6
Injury mechanism, no. (%)		
Traffic accidents	107 (55.7)	47 (45.6)
Falls	59 (30.7)	40 (38.8)
Other	26 (13.5)	16 (15.5)
GCS score at ED^b^	15 [15–15]	15 [15–15]
GOSE^b^	7 [6–8]	7 [6–8]
BAI score^a^	8.91 ± 9.32	7.51 ± 8.37
BDI score^a^	8.91 ± 7.94	8.09 ± 8.04
Antidepressant medication use	3 (1.6)	2 (1.9)
Anti-anxiety medication use	5 (2.6)	4 (3.9)
Hypnotic medication use	8 (4.2)	4 (3.9)

*IQR* interquartile range, *GCS* Glasgow Coma Score, *ED* Emergency department, *GOSE* Extended Glasgow Outcome Scale, *BAI* Beck Anxiety Inventory, *BDI* Beck Depression Inventory

^a^Mean ± standard deviation

^b^Median [IQR]

### Confounding factors for emotional symptoms following mTBI

The correlation between patient characteristics and BAI, BDI scores were analyzed using linear regression model to evaluate potential confounding factors. BAI and BDI scores showed moderate correlation with each other in the first and sixth week following mTBI (Additional file 1: Figure S1 and S2). The covariates including GCS, GOSE, injuries caused by traffic accidents or falls, and current antidepressant or hypnotic medication use were correlated with BAI and BDI (Additional file 1: Table S1). These covariates were adjusted for potential confounding effects.

### Association between BAI scores and the rs6265 polymorphism in the first week following mTBI

First, we examined whether rs6265 of the *BDNF* gene was associated with BAI scores. This questionnaire was completed in the first and sixth week after mTBI. Table 2 shows BAI scores among the three genotypes in the first week (TT genotype: 11.41 ± 1.88; CT genotype: 9.67 ± 1.20; CC genotype: 6.39 ± 1.35). Patients carrying the T allele showed significantly higher scores of BAI through Log-additive model (*p* = 0.028) in the first week. In addition, the male patients carrying the T allele also showed higher BAI scores in Log-additive model (*p* = 0.01). However, the BAI scores were no differences between the three genotypes in female patients.

**Table 2:** Association analysis between rs6265 polymorphism and BAI scores in the first week following mTBI

Genotype	Sample no. (%)	BAI score^a^	Codominant *p* value	Dominant *p* value	Recessive *p* value	Log-additive *p* value
Total (*n* = 184)						
TT	40 (21.7)	11.41 ± 1.88	0.083	0.117	0.043*	0.028*
CT	89 (48.4)	9.67 ± 1.20				
CC	55 (29.9)	6.39 ± 1.35				
Male (*n* = 57)						
TT	14 (24.6)	8.80 ± 1.97	0.037*	0.054	0.022*	0.010*
CT	24 (42.1)	6.15 ± 2.52				
CC	19 (33.3)	4.36 ± 1.56				
Female (*n* = 127)						
TT	26 (20.5)	12.94 ± 2.72	0.229	0.400	0.091	0.119
CT	65 (51.2)	10.81 ± 1.34				
CC	36 (28.3)	7.50 ± 1.89				

^a^Mean ± standard error (s.e.). The *p* value was adjusted for GCS, GOSE, injury mechanism, antidepressant medication use and hypnotic medication use. *p < 0.05 is labeled in bold

### Association between BAI scores and the rs6265 polymorphism in the sixth week following mTBI

BAI scores among the three genotypes in the sixth week (TT genotype: 6.64 ± 2.20; CT genotype: 7.85 ± 1.36; CC genotype: 8.21 ± 1.96) are shown in Table 3. There were no significant differences among the three genotypes. However, the male patients carrying the T allele still showed significantly higher BAI scores in Log-additive model (*p* = 0.038).

**Table 3:** Association analysis between rs6265 polymorphism and BAI scores in the sixth week following mTBI

Genotype		Sample no. (%)	BAI score^a^	Codominant *p* value	Dominant *p* value	Recessive *p* value	Log-additive *p* value
Total (*n* = 103)							
TT		16 (15.5)	6.64 ± 2.20	0.578	0.300	0.850	0.423
CT		55 (53.4)	7.85 ± 1.36				
CC		32 (31.1)	8.21 ± 1.96				
Male (*n* = 36)							
TT		5 (13.9)	10.33 ± 6.57	0.060	0.225	0.019*	0.038*
CT		20 (55.6)	6.36 ± 2.33				
CC		11 (30.6)	2.38 ± 1.00				
Female (*n* = 67)							
TT		11 (16.4)	5.25 ± 1.96	0.166	0.082	0.193	0.059
CT		35 (52.2)	8.59 ± 1.69				
CC		21 (31.3)	11.13 ± 2.62				

^a^Mean ± standard error (s.e.). The *p* value was adjusted for GCS, GOSE, injury mechanism, antidepressant medication use and hypnotic medication use. *p < 0.05 is labeled in bold

### Association between BDI scores and the rs6265 polymorphism in the first week following mTBI

Depressive symptoms were measured by BDI scores in the first week and sixth week after brain injury. BDI scores among the three genotypes in the first week (TT genotype: 12.56 ± 1.62; CT genotype: 8.83 ± 0.97; CC genotype: 7.06 ± 1.72) are shown in Table 4. Patients carrying the T allele showed higher scores of BDI (*p* = 0.006). In the gender-specific analysis, the female patients carrying the T allele had significantly higher BDI scores in the first week using log-additive model *(p* = 0.015). Meanwhile, the male patients carrying the T allele also showed higher BDI scores (*p* = 0.029).

**Table 4:** Association analysis between rs6265 polymorphism and BDI scores in the first week following mTBI

Genotype	Sample no. (%)	BDI score^a^	Codominant *p* value	Dominant *p* value	Recessive *p* value	Log-additive *p* value
Total (*n* = 186)						
TT	41 (22.0)	12.56 ± 1.62	0.009**	0.144	0.002**	0.006**
CT	89 (47.8)	8.83 ± 0.97				
CC	56 (30.1)	7.06 ± 1.72				
Male (*n* = 60)						
TT	15 (25.0)	8.10 ± 1.22	0.093	0.050	0.107	0.029*
CT	26 (43.3)	8.50 ± 2.09				
CC	19 (31.7)	6.36 ± 3.15				
Female (*n* = 126)						
TT	26 (20.6)	15.18 ± 2.25	0.007**	0.381	0.002**	0.015*
CT	63 (50.0)	8.95 ± 1.10				
CC	37 (29.4)	7.43 ± 2.09				

^a^Mean ± standard error (s.e.). The *p* value was adjusted for GCS, GOSE, injury mechanism, antidepressant medication use and hypnotic medication use. *0.01 ≤ *p* < 0.05 is labeled in bold. **p < 0.01 is labeled in bold

### Association between BDI scores and the rs6265 polymorphism in the sixth week following mTBI

BDI scores among the three genotypes in the sixth week (TT genotype: 11.55 ± 2.90; CT genotype: 9.30 ± 1.46; CC genotype: 6.83 ± 1.79) are shown in Table 5. There were no significant differences among the three genotypes. However, the male patients carrying the T allele showed significantly higher BDI scores in the sixth week (*p* = 0.021).

**Table 5:** Association analysis between rs6265 polymorphism and BDI scores in the sixth week following mTBI

Genotype	Sample no. (%)	BDI score^a^	Codominant *p* value	Dominant *p* value	Recessive *p* value	Log-additive *p* value
Total (*n* = 103)						
TT	16 (15.5)	11.55 ± 2.90	0.327	0.382	0.148	0.170
CT	55 (53.4)	9.30 ± 1.46				
CC	32 (31.1)	6.83 ± 1.79				
Male (*n* = 36)						
TT	5 (13.9)	14.00 ± 7.02	0.032*	0.174	0.010*	0.021*
CT	20 (55.6)	6.91 ± 2.56				
CC	11 (30.6)	2.13 ± 0.61				
Female (*n* = 67)						
TT	11 (16.4)	10.63 ± 3.29	0.878	0.620	0.767	0.621
CT	35 (52.2)	10.50 ± 1.76				
CC	21 (31.3)	9.19 ± 2.49				

^a^Mean ± standard error (s.e.). The *p* value was adjusted for GCS, GOSE, injury mechanism, antidepressant medication use and hypnotic medication use. *p < 0.05 is labeled in bold

## Discussion

In this study, we evaluated the role of genetic polymorphism of *BDNF* (rs6265) in the emotional symptoms in patients with mTBI. We recruited 192 mTBI patients to evaluate anxiety and depressive symptoms using the BAI and BDI scores in the first and sixth week after mTBI. Results from BDI scores showed a strong association between the rs6265 polymorphism and depressive symptoms following mTBI in the first week. Patients carrying the T allele had more depressive symptoms than patients carrying the C allele. In addition, BAI scores revealed that patients carrying the T allele also had more anxiety symptoms than patients carrying the C allele in the first week. However, the influence of rs6265 on anxiety and depressive symptoms in the sixth week was not observed in our study.

The gender-specific subgroup analyses revealed that the male patients carrying the T allele of rs6265 had higher scores of BAI and BDI in the both first and sixth week. The female patients carrying the T allele had significantly higher scores of BDI in the first week, whereas the difference was not shown in the sixth week. We speculate that this gender differences may be explained by the interaction between sex steroid hormones and *BDNF* following brain injury. Accumulating evidence indicates that estrogen regulates the *BDNF* expression through many several mechanisms such as methylation or directly bind to the *BDNF* gene via estrogen receptor [40, 44–46].

Our study reported an important role of BDNF in the emotional symptoms after mTBI. Indeed, recent genetic association studies indicated the correlations between rs6265 of *BDNF* and cognitive outcomes after mTBI [35, 36, 47]. The mechanisms of depressive disorders may be due to aberrant regulation of neuronal plasticity, including neurotrophic factors that regulate neurogenesis in the hippocampus and limbic system [19]. Previous animal studies also showed that deletion of *BDNF* increases susceptibility to depressive effects [48, 49], and hippocampus-specific knockdown of *BDNF* produces several depressive-like behaviors in rats [26]. Meanwhile, recent clinical studies indicated that BDNF might decrease the hippocampal volume and increase the risk of developing depression in those exposed to environmental stress or trauma [50–53]. These evidence provide possible mechanisms for the findings in our present study.

There are several limitations to our study. First, the small sample size and short study duration may limit our findings. Second, other genetic polymorphisms of the *BDNF* gene were not investigated in the present study. Conducting direct *BDNF* sequencing may be useful for identifying new SNPs in the *BDNF* gene and clarifying the effects of *BDNF* polymorphisms in various outcomes of mTBI. This study provides evidence for the correlation between genetic polymorphism of rs6265 of *BDNF* and emotional symptoms in the early phase after mTBI. Since our study duration range from one to sixth weeks, further studies with longer duration of follow-up are needed to elucidate the long term effects of the rs6265 polymorphism on emotional symptoms following mTBI.

## Conclusions

Our study provides evidence for the correlation between the *BDNF* variant rs6265 and emotional symptoms in the early phase after mTBI.

## Additional file

Additional file 1: Table S1. The confounding factors for BAI and BDI scores. **Figure S1.** Correlation between BAI and BDI score in the first week (*p*-value < 0.001; Adjusted R^2^ = 0.3101). **Figure S2.** Correlation between BAI and BDI score in the sixth week (*p*-value < 0.001; Adjusted R^2^ = 0.5013). (DOCX 175 kb)

## Abbreviations

APOE: Apolipoprotein E
BAI: Beck anxiety inventory
BDI: Beck depression inventory
BDNF: Brain-derived neurotrophic factor
CDC: Center for disease control and prevention
COMT: Catechol-O-methyltransferase
ED: Emergency department
GCS: Glasgow coma scale
GOSE: Extended glasgow outcome scale
IL-6: Interleukin 6
MAO-A: Monoamine oxidase A
mTBI: Mild traumatic brain injury
PCR: Polymerase chain reaction
SD: Standard deviation
SDS: Sodium dodecyl sulfate
SNP: Single nucleotide polymorphism
TBI: Traumatic brain injury
