# Supplementary material for: The association between BDNF Val66Met polymorphism and emotional symptoms after mild traumatic brain injury
Source: BMC Med Genet. 2018 Jan 22;19:13. doi: 10.1186/s12881-017-0518-0 (PMC5776765; doi:10.1186/s12881-017-0518-0)
Supplement: Additional file 1: Table S1. — The confounding factors for BAI and BDI scores. Figure S1. Correlation between BAI and BDI score in the first week (p-value < 0.001; Adjusted R2 = 0.3101). Figure S2. Correlation between BAI and BDI score in the sixth week (p-value < 0.001; Adjusted R2 = 0.5013). (DOCX 175 kb) [file 12881_2017_518_MOESM1_ESM.docx]

| **Table S1. The confounding factors for BAI and BDI scores** | | | | | | | | | | | | |
| --- | --- | --- | --- | --- | --- | --- | --- | --- | --- | --- | --- | --- |
|  | **BAI scores (first week)** | | | **BDI scores (first week)** | | | **BAI scores (sixth week)** | | | **BDI scores (sixth week)** | | |
| **Variables** | **Beta** | **Std. Err.** | ***P* value** | **Beta** | **Std. Err.** | ***P* value** | **Beta** | **Std. Err.** | ***P* value** | **Beta** | **Std. Err.** | ***P* value** |
| **Gender** | 2.721 | 1.476 | 0.0669 | 0.603 | 1.248 | 0.630 | 1.303 | 1.732 | 0.453 | 1.544 | 1.662 | 0.355 |
| **Age** | 0.068 | 0.046 | 0.13996 | 0.007 | 0.039 | 0.856 | -0.002 | 0.053 | 0.967 | -0.046 | 0.051 | 0.370 |
| **Traffic accidents** | -0.113 | 1.386 | 0.935 | -0.232 | 1.172 | 0.844 | 4.297 | 1.607 | **0.009*** | 3.205 | 1.565 | **0.043*** |
| **Falls** | -0.255 | 1.489 | 0.864 | -0.139 | 1.254 | 0.912 | -4.969 | 1.626 | **0.003*** | -3.781 | 1.589 | **0.019*** |
| **Other** | 0.702 | 2.009 | 0.727 | 0.753 | 1.710 | 0.660 | 0.871 | 2.285 | 0.704 | 0.785 | 2.195 | 0.722 |
| **GCS** | 2.644 | 6.168 | 0.669 | -6.808 | 3.207 | **0.035*** | -15.096 | 5.928 | **0.013*** | 2.926 | 5.876 | 0.62 |
| **GOSE** | -2.848 | 0.611 | **6.11E-06*** | -2.079 | 0.521 | **9.67E-05*** | -1.764 | 0.827 | **0.035*** | -1.097 | 0.815 | 0.181 |
| **Antidepressant medication use** | 1.669 | 4.441 | 0.708 | 9.591 | 3.715 | **0.011*** | 11.078 | 5.799 | 0.060 | 11.247 | 6.030 | 0.066 |
| **Anti-anxiety**  **medication use** | -2.508 | 4.437 | 0.573 | -3.353 | 3.807 | 0.38 | -0.239 | 4.265 | 0.955 | -2.710 | 4.418 | 0.542 |
| **Hypnotic**  **medication use** | 8.380 | 3.297 | **0.012*** | 7.959 | 2.675 | **0.004*** | 3.199 | 4.248 | 0.454 | 5.754 | 4.377 | 0.193 |
| BAI, Beck Anxiety Inventory. BDI, Beck Depression Inventory. Other, Sports-related injuries or workplace accidents. GCS, Glasgow Coma Score. GOSE, Extended Glasgow Outcome Scale. * *p* < 0.05 is labeled in bold. | | | | | | | | | | | | |

**
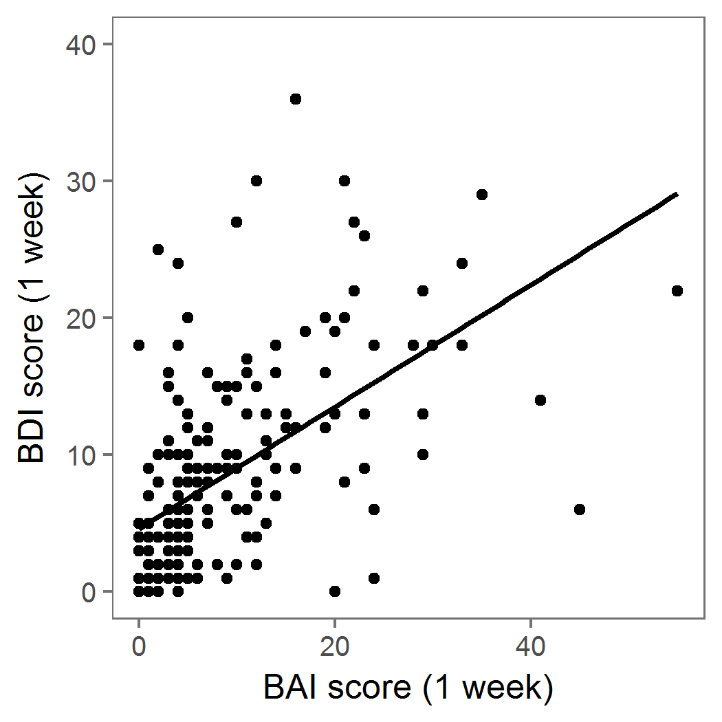
Figure S1. Correlation between BAI and BDI score in the first week (*p*-value < 0.001; Adjusted R^2^ = 0.3101)**


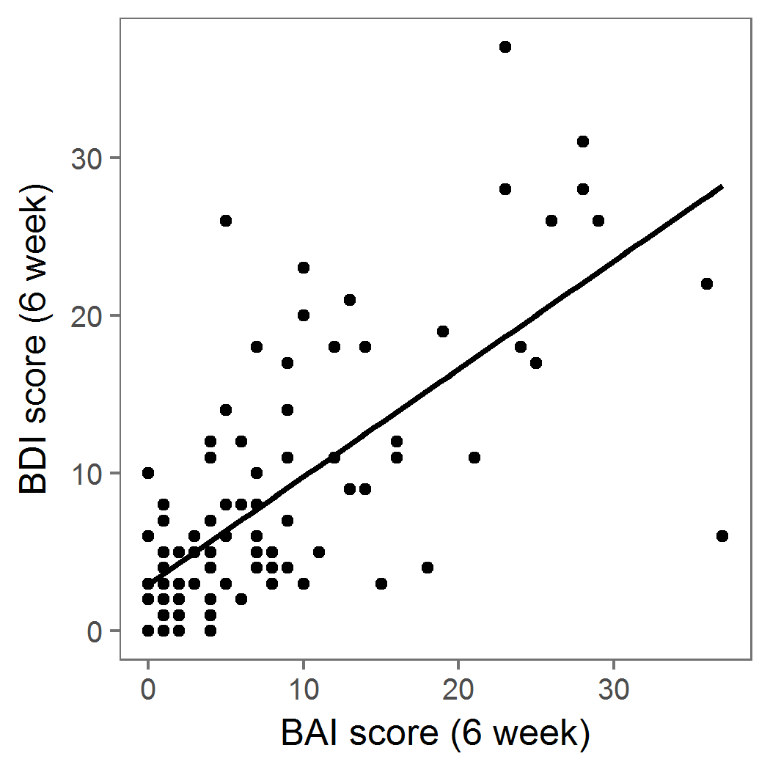


**Figure S2. Correlation between BAI and BDI score in the sixth week (*p*-value < 0.001; Adjusted R^2^ = 0.5013)**
